# Seed bank characteristics of the *Nymphoides peltata* population in Lake Taihu

**DOI:** 10.1038/srep13261

**Published:** 2015-08-18

**Authors:** Wei Huang, Qiuwen Chen, Kaining Chen

**Affiliations:** 1 CEER, Nanjing Hydraulic Research Institute, Nanjing 210029, China; 2 NIGLAS, Chinese Academy of Sciences, Nanjing 210008, China

## Abstract

The *Nymphoides peltata* (*N. peltata*) population has shown rapid expansion in Lake Taihu, China, in recent years. The core question is whether *N. peltata* seeds have contributed to the expansion. To address this, we randomly selected three *N. peltata* stands to investigate the seed bank characteristics of *N. peltata* in Lake Taihu. Results showed that *N. peltata* had high seed production, with a maximum seed yield of 1763 seeds per m^2^. Density of intact and fragmented seeds decreased rapidly with sediment depth. Few intact or fragmented seeds were distributed at depths greater than 4 cm in the sediment. Spatial distribution of the seed bank indicated that most seeds sank to the sediment within the *N. peltata* stands, and few seeds took advantage of their floating ability. Seeds recovered from the sediment during April to June had a low germination rate, and no seeds germinated during October to April. Cold exposure treatment increased the germination rate remarkably. No seedlings were found in the field from January 2012 to December 2012, indicating that few seeds were successfully established in the surveyed area. The results suggested that sexual reproduction had little direct contribution to the *N. peltata* expansion in this large shallow lake.

*Nymphoides peltata* is a common and relatively widespread aquatic plant^1^. In the past few decades, *N. peltata* has become an invasive pest species in North America and New Zealand^2^,^3^. In China, this native species has rapidly expanded in waterways and lakes, such as Lake Taihu in Jiangsu Province. This species tends to grow in dense patches^4^,^5^, which not only threatens recreational vessels, but also lead to stagnant areas with low oxygen levels^6^ and the competitive exclusion of other submerged macrophytes with overwhelming superiority^7^.

*Nymphoides peltata* propagates by vegetative and sexual means^8^. The small-sized seeds can float on the water surface, which should contribute to long distance dispersal. The ecology of *N. peltata* seeds has been studied due to the quick expansion of *N. peltata* populations in many lakes. Previous research has described development progress from the flower bud to fruit, with the mean number of developed seeds per m^2^ calculated to be 9434 in an experimental tank (S.D. = 5668, n = 4)^9^. However, studies on the production of fruits and seeds in the field remain limited^10^,^11^. When fruits are well developed, seeds are released and float on water by holding a hydrophobic surface^8^, which could be beneficial for efficient dispersal. Van der Velde and Van der Heijden^9^ reported that seeds remained floating in undisturbed Petri dishes after two months, though other studies have shown that most seeds were immediately submerged under the interference of simulated rain^12^. How far *N. peltata* seeds can float in the field remains unclear, and knowledge on the spatial distribution of seed density in sediment inside and outside wild *N. peltata* population stands is also limited.

After being shed from parent plants, mature seeds eventually settle to the soil surface and form a seed bank^13^. Viable seeds present by the end of the germination period and the period of seed release is considered a persistent seed bank^12^,^14^,^15^. In an unpredictable environment, a persistent seed bank is a mechanism to buffer the effect of environmental variability^13^, and thus the existence of a persistent seed bank prevents the risk of extinction through random fluctuations and plays an important role in population recruitment^16^. Although *N. peltata* has expanded quickly and has many negative effects on the native ecosystem, little work had been undertaken on the seed bank characteristics of *N. peltata*, which is essential for better understanding the contribution of sexual recruitment to the expansion of *N. peltata* populations.

Seed germination is a vital step in the process of population establishment. Takagawa *et al.*^17^ reported that safe-sites for *N. peltata* seed germination were less prone to inundation, and were exposed to sufficient light during the spring water-level drawdown. However, seeds located far from the shoreline might be faced with different conditions, such as low oxygen concentration and high sediment deposition rate. After seed germination, successful seedling establishment is also important for population recruitment.

In this paper, we hypothesized that sexual reproduction may play an important role in the expansion of the *N. peltata* population in the open waters of Lake Taihu. Thus, several aspects were investigated in Lake Taihu, including (1) seed yields; (2) spatial distribution of the seed bank inside and outside *N. peltata* population stands; (3) annual dynamics of the seed bank; and (4) seed germination and seedling density in *N. peltata* stands. These experiments were expected to provide a detailed understanding on the function of sexual recruitment in *N. peltata* population expansion in Lake Taihu.

## Results

### Seed yield

The reproductive organ development patterns differed between the A, B and C communities (Table 1). Flower density and bud density per inflorescence were larger in B and C than in A, while the fruit density per inflorescence was largest in A. This indicated that stand A flowered earlier than the other two stands. There was also great variation in the maximum seed yield per m^2^ among the three stands, with a range of 428 to 1763 seeds per m^2^.

### Spatial and temporal distribution of the seed bank

**Figure 1:**
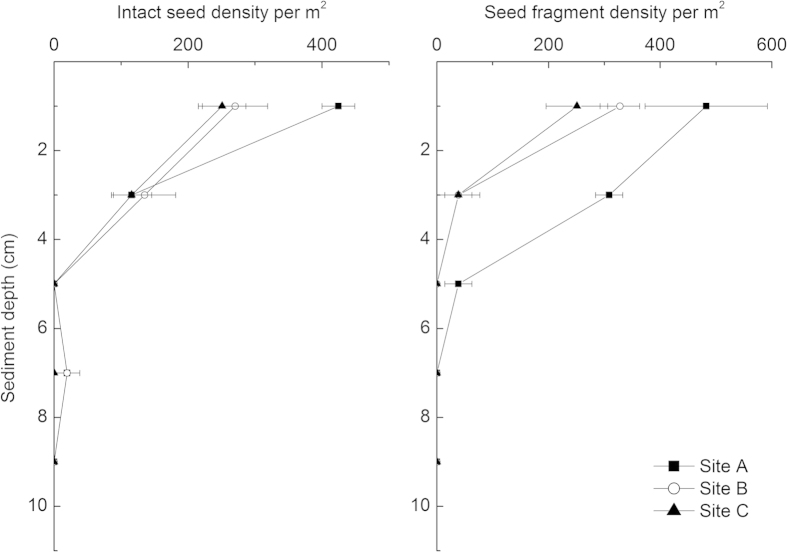
Seed density of *Nymphoides peltata* with sediment depth in Lake Taihu in September 2011 (means ± S.D. are shown).

Vertical distribution of seeds at the three sites showed a similar pattern (Fig. 1). The *N. peltata* seeds were mainly distributed on the sediment surface in September 2011. The number of intact and fragmented seeds decreased rapidly with sediment depth. Few intact seeds or seed fragments were distributed at depths greater than 4 cm in all three *N. peltata* stands. These results suggested that a sediment depth up to 10 cm would accurately determine *N. peltata* seed density in this study area.

**Figure 2:**
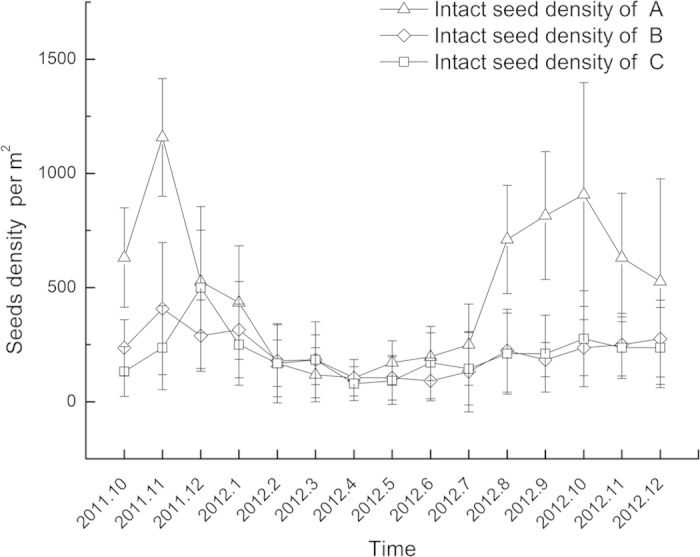
Temporal dynamics (2011.10–2012.12) of intact seed density in three *Nymphoides peltata* stands (means ± S.D. are shown).

**Figure 3:**
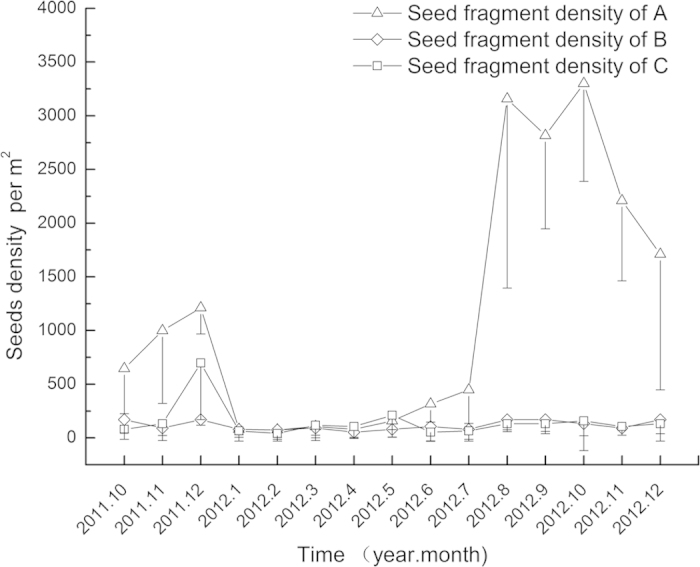
Temporal dynamics (2011.10–2012.12) of fragmented seed density in three *Nymphoides peltata* stands (means ± S.D. are shown).

As seen from Figs 2 and 3, considerable variation existed in seed banks between the three stands. However, the seasonal dynamics of seed banks had a similar feature. The *N. peltata* seed bank increased from October 2011 to November 2011, then decreased gradually from December 2011 to March 2012, and reached to a relatively steady level of 123 ± 101 (mean ± S.D., n = 27) seeds per m^2^ in May 2012. After May 2012, however, the seed bank increased rapidly, with a maximum of 908 ± 490 (mean ± S.D., n = 10) seeds per m^2^. The temporal variation in seed fragment density in the present study was similar to that of intact seeds.

**Figure 4:**
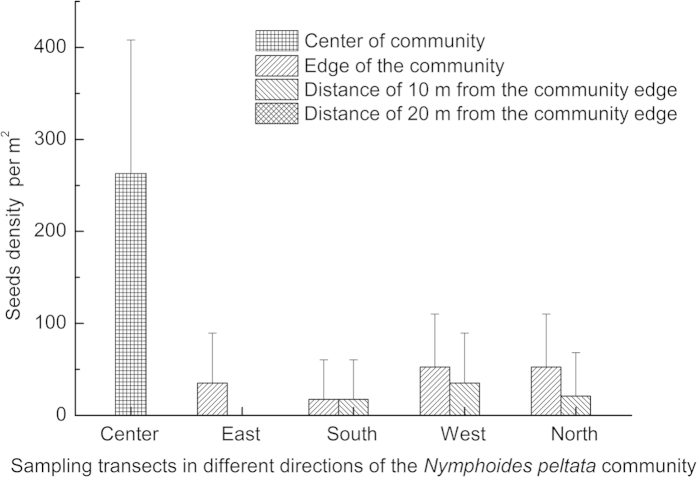
Spatial distribution of *Nymphoides peltata* seeds in sediment of stand B (means ± S.D. are shown).

Seed density had a maximum value of 263 ± 145 seeds per m^2^ in the center of the community of stand B, and decreased sharply along the transects (Fig. 4). The seed density in the center was six times that of the density at the edge of the community. Seed density was low in sites 10 m from the community edge. No seed was found in sediment cores in sites 20 m from the edge of the *N. peltata* community.

### Seedling dynamics and germination

From January 2012 to December 2012, seedling densities were estimated monthly by counting seedlings in the field. However, no *N. peltata* seedling was found when investigating the seed bank in the field.

The germination rate of seeds recovered from the sediment was low and decreased with time. Seed germination rates were 21.1%, 18.9% and 11.1% in April, May and June, respectively. Cold exposure treatment increased the germination rate remarkably. Cumulative germination rates were 73.3%, 74.4% and 76.7% in April, May and June, respectively. Seed bag experiments showed that no seed germinated below or above the sediment in Lake Taihu. Non-germinated seeds were stained with tetrazolium, which showed they were viable.

## Discussion

The sizes of the three investigated stands were similar with a radius of around 60 m. The coverage of stands A, B and C were 100%, 100% and 80%, respectively. Accompanying species were *Vallisneria natans* and *Hydrilla verticillata* in site A and B, *Potamogeton malaianus* in site C. Great differences at reproductive stages were observed between the three *N. peltata* stands in Lake Taihu. *N. peltata* flowered earlier in site A than in sites B and C. One possible reason was the difference in sediment type: very dark silt in site A compared with shallow silt-loam in sites B and C. Sediment is considered as the main nutrient source for rooted macrophytes^18^,^19^, and can affect macrophyte development. In Nijmegen (Netherlands), Van der Velde and Van der Heijden^9^ reported that the mean number of fruits per m^2^ was 180 (S.D. = 76.3, n = 25), with an average of 26.5 seeds per fruit. They also calculated the average number of developed seeds per m^2^ to be 3117 in a *N. peltata* stand. The results of the present study revealed that *N. peltata* had a smaller seed number per m^2^ in Lake Taihu, probably due to the different climate and eutrophic level of water.

Seeds were found mostly concentrated in the top sediment layer, in agreement with previous studies^20^,^21^. This is possibly due to seed longevity. Thompson^22^ suggested that small, round compact seeds are persistent in soil, while large, flat seeds are often short lived. Since the seeds of *N. peltata* are flat, broad-elliptical and thick^4^,^9^, they may be transient in sediment. Therefore, seed density decreased sharply with sediment depth.

The temporal pattern of intact seed density in sediment indicated that many fresh seeds fell to the sediment and become part of the seed bank from October 2011 to November 2011. After that, the seed number experienced a net reduction from December 2011 to May 2012, which was probably caused by seed emergence and feeding by animals^8^. After May 2012, reproductive organs began to develop, followed by many seeds being released from mature fruits and settling to the sediment surface. In addition, seeds would not germinate without exposure to cold temperature^11^ and seed consumption by animals may be limited^8^, which led to the rapid increase in seed density from May 2012. Temporal dynamics of seed fragment density was similar to that of intact seed density, which indicated that most seed fragments decomposed rapidly. As seen in Fig. 2, a persistent seed bank occurred in *N. peltata* stands, which is vital for population recruitment^13^. Existence of a persistent seed bank prevents the risk of extinction through unexpected disasters^16^. It is supposed that it takes several years to exhaust seed banks through seedling emergence^23^. If *N. peltata* cannot produce seeds under poor environmental conditions and few asexual propagates are conserved, sexual reproduction may recruit the population and improve the stability of the *N. peltata* community.

Dispersal ability plays an important role in population dynamics. Van der Velde and Van der Heijden^9^ evaluated the floating ability of *N. peltata* seeds, and reported that *N. peltata* seeds remained floating in Petri dishes for two months without any disturbance. However, the results of spatial distribution in the present study indicated that most seeds sank quickly to the sediment within the *N. peltata* stands. One possible reason might be that *N. peltata* populations usually form dense patches with high leaf index area in lakes^10^. When ripe seeds are released from fruits, the floating seeds may encounter numerous leaves on the water surface, which may break the hydrophobic coating outside the seeds^8^. As a result, seeds would sink quickly within the stand. Seeds released at the edge of the stand are more likely to be the source of long-distance dispersal.

The results showed evidence that few *N. peltata* seedlings were established in the study area of Lake Taihu. Previous studies also suggest that only a small area of the lake has safe sites for *N. peltata* seedling establishment^17^,^24^. Even if some *N. peltata* seeds can germinate, most detach from the sediment and float to the water surface^4^,^25^.

Seed germination experiments revealed that only a small proportion of seeds germinated readily when collected from sediment during April to June. Seed dormancy can be divided into physical and physiological (innate) dormancy. The geminated seeds may be categorized as physically dormant^26^, since *N. peltata* seeds were unable to germinate when they were under hypoxic conditions in the sediment^4^,^11^. Most of the non-germinated seeds were innately dormant, with four weeks cold exposure overcoming their physiological dormancy, indicating that the natural cold exposure period was not long enough in winter and further cold treatment was needed to break seed dormancy in the following year in Lake Taihu. The non-germinated seeds were gradually covered by sediment until the next year, and physiologically dormant seeds probably became physically dormant, which might inhibit successful germination.

Previous studies indicate that *N. peltata* expands locally with runners. If the runners have developed roots that are not yet attached to the sediment and the runners are broken by natural forces, vegetative fragments will be released and eventually dispersed to favourable areas where they can establish themselves^3^,^9^. Recently, Larson used molecular markers to investigate reproduction strategy of *N. peltata* in Sweden^27^. Lacking of genetic variation suggested that vegetative reproduction constituted an important part of the total reproduction^27^. These literatures indirectly confirm that sexual reproduction may play a minor role in population expansion.

## Conclusion

This study suggested that *N. peltata* could produce a large number of seeds in Lake Taihu. Most seeds sank within the stand and a large proportion of the seeds were innately dormant, with no seedlings found in the study area. Sexual reproduction contributed little to the rapid expansion of *N. peltata* in the open waters of this large shallow lake. The expansion of *N. peltata* populations was probably maintained by the dispersal of vegetative fragments.

## Methods

### Study site

**Figure 5:**
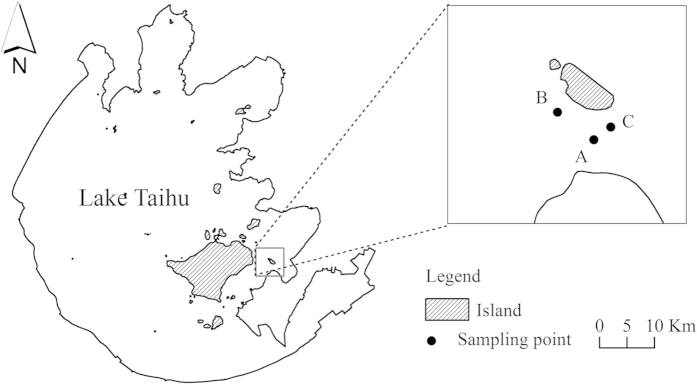
Study area and sampling points of Lake Taihu (created by ArcGIS 10.0, Environmental Systems Research Institute, Inc., Redlands, CA, USA).

Lake Taihu is a shallow lake with a surface area of 2,338 km^2^ and a mean depth of 1.9 m. In the 1950s, the lake was oligotrophic. Since the 1980s, however, water quality has continuously deteriorated and the lake is now eutrophic. Blue-green algae dominate the western part of the lake, whilst the eastern part is mainly covered by vascular plants^5^,^28^. Satellite images of Lake Taihu at different periods revealed that the population of hydrophytes has been increasing rapidly since 2001 and floating-leaved macrophytes dominate the lake, covering a surface area of 89.1 km^2^ in 2004^5^. According to field surveys conducted in the summers of 2004 and 2013, *N. peltata* is the dominant floating-leaved macrophyte in Lake Taihu^4^. To investigate the seed characteristics of *N. peltata*, three *N. peltata* stands were carefully selected for our study (Fig. 5), which were denoted as A (31.12070° N, 120.39241° E), B (31.12295° N, 120.38566° E) and C (31.12460° N, 120.39346° E). In each stand, *N. peltata* has been colonized for more than ten years. The three *N. peltata* stands we chose were isolated to each other, and the shapes of the three stands were almost round; while most other stands were closed to each other, making it difficult to investigate the spatial distribution of seed and to assess the dispersal ability. In addition, according to our previous field survey conducted in the summer of 2004, 2008 and 2010, most sediment colonized by *N. peltata* was silt. The sediments of site A, B and C were all silt. Therefore, It was representative to choose the three sites as the study place.

### Estimation of seed yield

The field investigation showed that the growing season of *N. peltata* ranged from April to late October. Blooming began in late May or early June. Peak blooming occurred from August to October, and a few flowers continued emerging until November in Lake Taihu. This was similar to the flowering period reported in the Netherlands^9^. To estimate the seed yield of *N. peltata*, 15 quadrats (1 m^2^) were randomly established in the three stands on September 1, 2012. The inflorescence number in each quadrat as well as fruit, flower and flower bud numbers per inflorescence were recorded. The average seed number of 40 randomly selected fruits from each community was counted. Maximum seed yield per m^2^ was estimated by multiplying the sum of bud number, flower number, fruit number, and inflorescence number, and average seed number.

### Characterizing spatial-temporal distribution of the seed bank

Seed separation was used to examine the *N. peltata* seed bank. Seed separation utilizes the differences in size or density to separate seeds from sediment^29^. Since the mean length and width of the *N. peltata* seeds are 3.8–5.1 mm and 2.7–3.0 mm, respectively^9^, it was possible to detect seeds efficiently from the sediment. In the study, the seeds were classified into two categories: intact seeds and seed fragments. Intact seeds were defined as seeds having intact and hard seed capsules with or without marginal bristles^12^; seed fragments included fragmented seed capsules with marginal bristles and decayed seeds.

The vertical distribution of the seeds was first investigated to estimate the *N. peltata* seed bank in Lake Taihu. Three replicate sediment cores (diameter 10 cm, length 25 cm) were taken randomly in the centers of the three *N. peltata* stands on September 1, 2011. Cores were immediately sliced into 2 cm sections from the surface to a 20 cm depth. Each section was put into a plastic bag and transported to the laboratory. The sediments were concentrated by washing with a gentle water flow through a fine sieve (0.425 mm mesh), and both the stone roots and vegetative parts were removed. The mesh was small enough to catch *N. peltata* seeds. The seeds were then selected using the seed separation method.

The spatial distribution of *N. peltata* seeds was investigated to reveal seed floating capacity. Stand B was chosen as the study site because the *N. peltata* community had a regular circle shape with a radius of 60 m, and there were no other *N. peltata* stands within 200 m. Sampling transects were made in four directions (east, south, west and north) from the center of *N. peltata* community. Field investigation showed that there was no seed in sediment 30 m from the edge of the community. Therefore, the farthest sample point was 20 m from the community edge. In addition, there were two other sampling points in each transect located at the edge of the community and 10 m from the community edge. Sediment cores were collected along established transects from the center of the *N. peltata* stand B. Nine replicate sediment cores (diameter 10 cm, length 25 cm) were taken in October 2012. The seed selection procedure followed the method described above.

### Estimation of seedling dynamic and germination

Seedling densities in the field were estimated by counting seedlings monthly from January to December 2012. An Ekman sediment sampler (15 cm × 15 cm × 20 cm) was employed to examine the density of the seedlings. Nine replicate samples were randomly taken in each of the three *N. peltata* stands. A water depth of approximately 10 cm was maintained over the sediment surface during the sampling process to avoid any disturbance of the sediment. The *N. peltata* seedlings were examined and counted.

The seeds picked from sediment cores in sites A, B and C from April to June in 2012 were used for the seed germination experiment. Seeds collected from the three communities were well mixed. Batches of 30 seeds were used and experiments were executed in triplicate. Seeds were incubated in a Petri dish filled with fresh tap water (0.3 mm depth) in which all seeds were submerged. Light was provided by a fluorescent white tube. Each treatment was tested at 20 μEinstein m^−2^ s^−1^ with a photoperiod of 14 h. All experiments were conducted at 25 °C in a room for a period of 4 weeks. Germinated seeds were counted and removed every other day and tap water was replenished weekly. Another experiment was conducted to evaluate the effect of cold stratification (here defined as storage in demineralized water at 4 °C) on the seed germination rate. After incubation at 25 °C for 4 weeks, the remaining non-germinated seeds were stored in a refrigerator for another 4 weeks at 4 °C. The seeds were then incubated in the same conditions as above. During the experiment, if the radicle protruded at least 1 mm from the seed, the seed was scored as germinated.

To study the seed germination process in the field, seed bag experiments were performed. Seeds were collected from mature fruits and placed in nylon mesh bags (0.5 mm mesh, 4.0 cm × 5.0 cm) on October 10, 2012. Each bag contained 50 seeds. Five bags were attached to an iron peg, which was pushed into the sediment to keep the seed bags buried at a depth of approximately 2 cm. To mimic the aboveground seed bank, the five bags were connected to the iron peg by a nylon line, and each bag contained a float ball to avoid the bags being covered by sediment. In May 2013, these bags were harvested to evaluate germination. To examine the viability of the seeds that did not germinate, the seeds were cut and the embryos were treated with tetrazolium solution^30^.

## Additional Information

**How to cite this article**: Huang, W. *et al.* Seed bank characteristics of the *Nymphoides peltata*population in Lake Taihu. *Sci. Rep.* **5**, 13261; doi: 10.1038/srep13261 (2015).

## Figures and Tables

**Table 1:** Investment in sexual reproduction in *Nymphoides peltata* in Lake Taihu.

**Sample site**	**Inflorescence density per m^2^**	**Fruit density per inflorescence**	**Flower density per inflorescence**	**Bud density per inflorescence**	**Seed density per fruit**	**Maximum seed yields per m^2^**
**A**	4.1 ± 0.6a	6.9 ± 3.0a	1.5 ± 1.3a	1.5 ± 1.7a	43.3 ± 7.8a	1763.9 ± 690.2a
**B**	2.3 ± 0.6b	6.6 ± 6.1a	3.1 ± 2.5b	7.4 ± 4.8b	39.8 ± 7.2a	1572.2 ± 755.5a
**C**	1.1 ± 0.6c	3.9 ± 3.2b	2.0 ± 1.0a	5.1 ± 3.1c	34.6 ± 6.5c	428.8 ± 142.2b

Means ± standard errors are presented. Values in a given row with different lowercase letters are significantly different (*P* < 0.05) according to Tukey’s honestly significant difference test (one-way ANOVA).
